# Awake transoral intralaryngeal botulinum toxin injection for early bilateral vocal fold immobility: a five-year experience

**DOI:** 10.1007/s00405-026-10430-6

**Published:** 2026-07-19

**Authors:** Reham Abdelwakil Ibrahim, Mohamed Mahmoud Mohamed Roushdy, Shereen Farghaly, Mohamed Modather Aboshanif

**Affiliations:** 1 Phoniatrics Unit, Otorhinolaryngology Department, Faculty of Medicine, Assiut University, Assuit, 71515 Egypt; 2 Otorhinolaryngology Department, Faculty of Medicine, Assiut University, Assiut, 71515 Egypt; 3 Department of Chest Diseases, Faculty of Medicine, Assiut University, Assiut, 71515 Egypt

**Keywords:** Bilateral vocal fold immobility, Botulinum toxin A, Airway obstruction, Staged injection, Tracheostomy avoidance

## Abstract

**Purpose:**

To describe a single-centre clinical experience with awake intra-laryngeal botulinum toxin type A (BTXA) injection in adults with early-onset bilateral vocal fold immobility (BVFI), and to report temporal clinical observations associated with a staged bilateral injection protocol.

**Methods:**

This retrospective observational study included 47 adult non-tracheostomized patients with early BVFI treated between January 2020 and March 2025. All patients underwent awake trans-oral BTXA injection under indirect laryngoscopic visualization (5 U per vocal fold). Bilateral treatment was performed using staged unilateral injections administered 24 h apart. Peak inspiratory flow (PIF) and Dyspnea Index (DI), voice and swallowing outcomes were assessed before injection and during follow-ups.

**Results:**

Improvement in airway-related measures was observed during follow-up. Median DI decreased after injection and median PIF increased during the expected pharmacological window of BTXA activity, with partial symptom recurrence by four months. Clinical improvement followed the known delayed pharmacodynamic profile of BTXA, with onset occurring after several days and peak effect within the first two weeks. Two patients required temporary tracheostomy and were subsequently decannulated. Voice and swallowing function remained largely preserved.

**Conclusions:**

Awake intra-laryngeal BTXA injection was feasible as a reversible temporizing intervention for selected patients with early bilateral vocal fold immobility during the observation period. The staged bilateral injection strategy is presented as an experience-based technical approach that might help avoid tracheostomy in selected patients; however, a 24-hour interval between injections didn’t appear to produce a clearly distinct staged pharmacodynamic effect. Findings should be interpreted as descriptive clinical observations requiring prospective validation.

**Level of evidence:**

4

## Introduction

Bilateral vocal fold immobility (BVFI) is a potentially life-threatening disorder characterized by absent or impaired vocal fold motion, most commonly due to recurrent laryngeal nerve (RLN) injury or, less frequently, mechanical fixation of the cricoarytenoid joint [1]. Resulting glottic narrowing causes airway obstruction, the severity of which depends on the paralyzed fold position, extent of nerve injury, and development of synkinesis [2]. Aberrant reinnervation of adductor muscles during RLN recovery can further worsen airway compromise [3]. Spontaneous nerve regeneration may occur within four to five months which is known as the observation period, during which irreversible procedures are often deferred [4]. Although tracheostomy secures the airway, it imposes substantial physical, cosmetic, and psychosocial burdens [5]. Many patients instead restrict daily activity, highlighting the need for minimally invasive, temporizing interventions that preserve airway patency and laryngeal function during this recovery phase.

Chemodenervation with botulinum toxin type A (BTXA) offers a temporary, minimally invasive alternative by weakening hyperactive adductor muscles and permitting unopposed abductor function to widen the glottis. BTXA produces transient paralysis by blocking acetylcholine release at the motor end plate [6, 7]. Prior studies support its role in relieving stridor in BVFI, though most are limited by small sample sizes. Intra-laryngeal BTXA injection is traditionally performed via a transcutaneous approach, with thyroarytenoid (TA) muscle localization guided by electromyography (EMG) [8–11]. Alternatively, indirect laryngoscopy using a curved needle allows direct visualization of the TA, the principal body of the vocal fold, and enables precise infiltration of adjacent intrinsic laryngeal muscles [12]. Filho and Rosen [11] demonstrated that unilateral injection of higher toxin doses can effectively enlarge the airway. However, the optimal timing and administration strategy for bilateral BTXA injection in early BVFI remain uncertain. In the present study, BTXA injection was performed under indirect laryngoscopic visualization using a staged bilateral injection protocol with unilateral injections administered 24 h apart. This study aimed to describe the clinical course and airway-related observations associated with this institutional practice during the early recovery period of BVFI.

This study focused on early-onset BVFI, typically within 4–5 months of paralysis onset. The primary aim of this study was to describe clinical observations following awake trans-oral intra-laryngeal BTXA injection as a temporizing intervention for airway symptoms during the observation period of early BVFI, based on a five-year institutional experience at a tertiary academic referral centre. A secondary aim was to report temporal clinical observations associated with a staged bilateral injection protocol administered 24 h apart.

## Materials and methods

### Study design and participants

This retrospective observational cohort study included adult patients diagnosed with bilateral vocal fold immobility (BVFI) by laryngoscopy between January 2020 and March 2025 at a tertiary academic referral centre. In our institutional practice, selected patients presenting in the early phase of BVFI (approximately within 4–5 months from symptom onset), a period during which spontaneous nerve regeneration may still occur, and experiencing exertional inspiratory stridor, were treated with intra-laryngeal botulinum toxin type A (BTXA) injection as part of their individualized clinical management. Treatment decisions were made on a case-by-case basis according to clinical severity and airway symptoms. For the purpose of this analysis, only patients who received BTX-A injection prior to any surgical airway intervention were included.

Exclusion criteria included cricoarytenoid joint fixation, laryngeal malignancy or trauma, co-existing respiratory disease, neurological disorders affecting respiratory function, and psychiatric conditions interfering with reliable symptom assessment. Extracted variables included age, sex, aetiology of BVFI, duration since symptom onset, and BTXA injection parameters (dose, number of injections, and technique). The study was approved by the Institutional Review Board of Assiut University Faculty of Medicine (IRB No. 04-2024-300442) and conducted in accordance with the Declaration of Helsinki. Written informed consent was obtained for all clinical procedures and for anonymised use of endoscopic data for research and publication.

### Voice, and swallowing evaluation

Voice quality was assessed by an experienced laryngologist blinded to clinical data using the modified GRBAS scale based on sustained /ah/ phonation recorded in a sound-treated room [13]. Swallowing function was evaluated using flexible endoscopic evaluation of swallowing (FEES) and graded using the Penetration-Aspiration Scale (PAS) [14].Patient-reported outcomes included the Arabic Voice Handicap Index-10 (VHI-10) and Dysphagia Handicap Index (DHI), where higher scores indicate greater perceived impairment [15, 16].

### Airway evaluation

Spirometry (Cosmed SrL, Quark PFT Ergo, Italy) was performed in all patients using standardised oral technique with nasal occlusion. Peak inspiratory flow (PIF, L/s) was recorded as a physiological indicator of upper airway patency and extrathoracic airflow limitation [17]. Dyspnoea severity was assessed using the Arabic Dyspnoea Index (DI), a validated patient-reported outcome measure for upper airway obstruction [18]. All measurements were used to describe longitudinal physiological and symptomatic changes following treatment.

### Botulinum toxin injection technique

A staged bilateral injection protocol, with unilateral injection administered 24 h apart was adopted as a pragmatic procedural modification based on prior institutional experience and concerns regarding transient airway compromise following simultaneous bilateral injection [19]. All procedures were performed by the same physician in the outpatient clinic with patients awake, seated upright, and the head slightly extended. Endoscopic visualization and needle placement were achieved using a 70° rigid laryngoscope (Hopkins^®^) connected to a TELE PACK X LED TP 100 system.

**Fig. 1:**
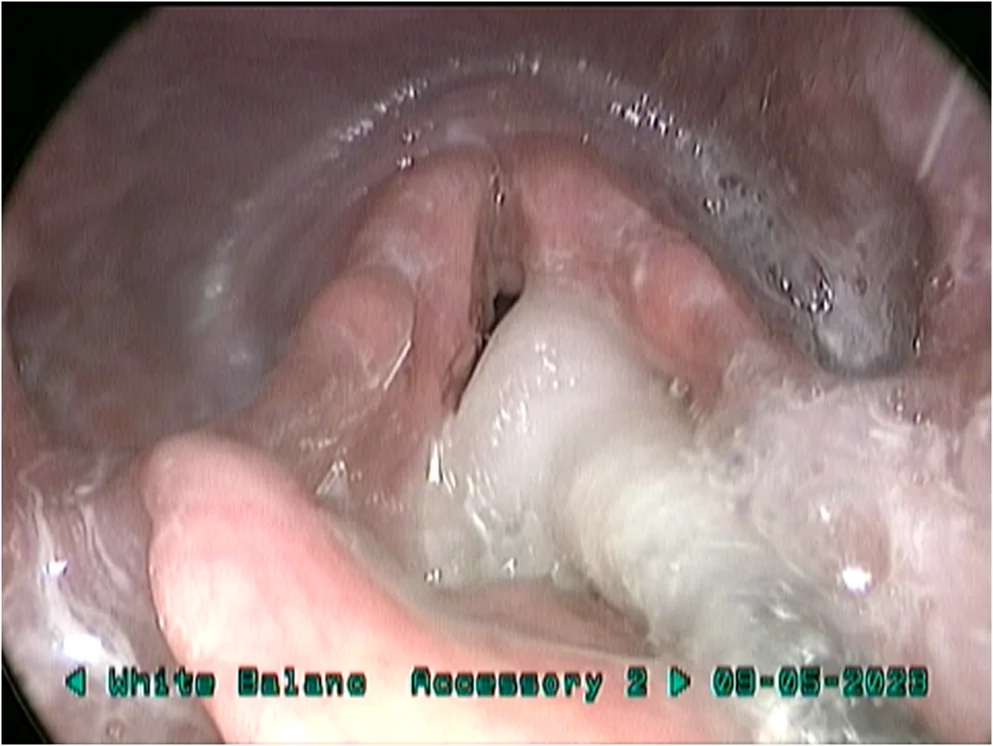
Palpation of the vocal fold with a curved laryngeal cotton applicator to confirm laryngeal anaesthesia. All images are anonymized and used with patient consent. Embedded text reflects endoscopic system metadata from the original capture

Anaesthesia: Topical anaesthesia was achieved using 4% lidocaine sprayed into the nasal cavity, oropharynx, and tongue base, followed by application of 0.5 mL over the supraglottic and glottic structures during phonation via the working channel of a flexible laryngoscope to facilitate dispersion. A curved laryngeal applicator (PELTESON, Karl Storz), wrapped in cotton soaked in a 1:1 solution of 4% lidocaine and ephedrine, was used to ensure adequate anaesthesia, reduce the risk of laryngospasm, and assess arytenoid mobility to exclude cricoarytenoid joint fixation (Fig. 1).

**Fig. 2:**
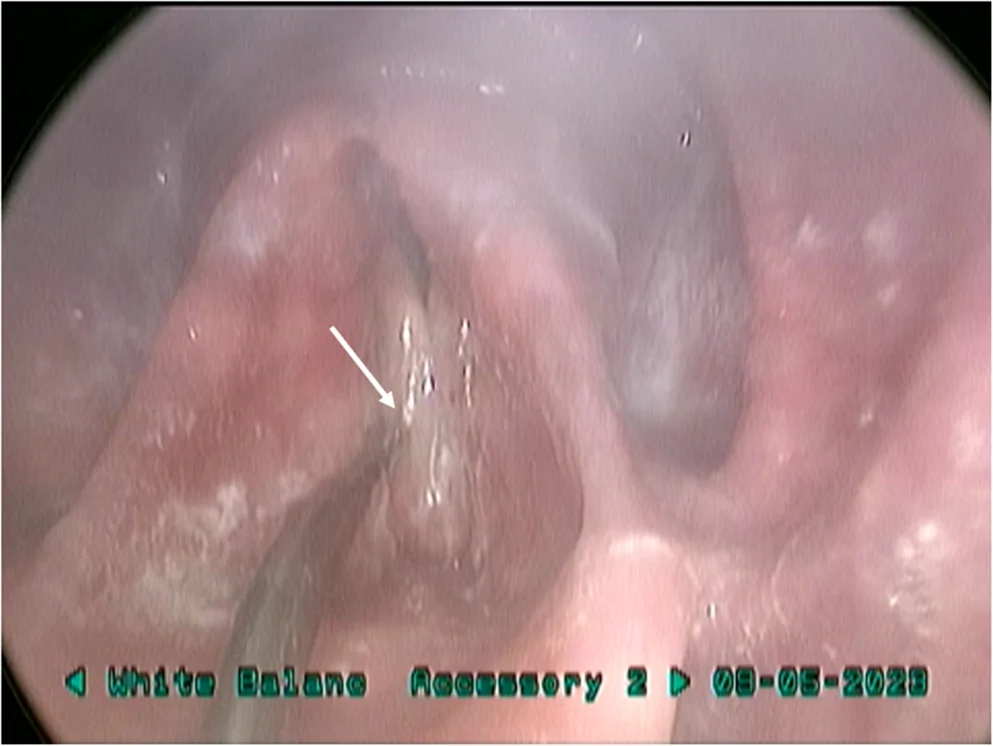
Laryngoscopic view showing the needle introduction at the injection site (white arrow). All images are anonymized and used with patient consent

Toxin preparation and injection: Botulinum toxin type A (BOTOX^®^, Allergan, Inc.) was reconstituted (100 U vial in 4 mL 0.9% saline; 2.5 U per 0.1 mL). Patients were instructed to perform gentle inhalation during procedure. Medial vocal fold bulging and arytenoid medial rotation during inspiration were used as indicators of synkinetic activity of the thyroarytenoid (TA) and lateral cricoarytenoid (LCA) muscles [20]. Using a curved laryngeal cannula (JOUSSEN, Karl Storz), BTX-A was injected into the middle third of the vibratory portion of the vocal fold, approximately 1 mm lateral to the junction between the vibratory portion and the floor of the laryngeal vestibule, to facilitate diffusion into both the TA and LCA muscles (Fig. 2). This injection site has been anatomically validated by Alonso et al. [21]. The total dose administered was 10 U (5 U per vocal fold), consistent with our institutional protocol [19]. All patients underwent unilateral injection, with 5 U administered to one vocal fold followed by contralateral injection 24 h later.

Post-injection care and follow-up: Following injection, patients received nebulized salbutamol (1 mg in 0.9% saline), humidified oxygen, and intravenous dexamethasone (8 mg) to minimize airway oedema or spasm; a second 8 mg dose was administered after contralateral injection when clinically indicated. Respiratory status was monitored for 24 h. Patients were discharged after 2 days with instructions regarding chin-down swallowing, symptom monitoring, and scheduled follow-up visits at 1, 2, and 6 weeks, and at 4 months. Patients who developed recurrent respiratory symptoms during or beyond follow-up were offered repeat injections. For the purpose of the present analysis, only outcomes within four months following the initial injection were included, corresponding to the expected pharmacological duration of BTXA activity; long-term airway outcomes beyond this period were not assessed.

#### Statistical analysis

Data were analysed using SPSS software (version 20; IBM Corp., Armonk, NY, USA). Given the retrospective observational design, relatively small sample size, and clinical heterogeneity of the cohort, analyses were considered exploratory and primarily descriptive. Continuous variables are presented as mean ± standard deviation (SD) or median and interquartile range (IQR), as appropriate, while categorical variables are presented as frequencies and percentages. Within-patient comparisons between baseline and follow-up assessments were performed using the Wilcoxon signed-rank test. Comparisons between independent subgroups (bilateral vocal fold paralysis versus paresis) were performed using the Mann–Whitney U test. Effect size (r) was calculated for Wilcoxon signed-rank analyses using the formula Z/√N. A two-sided p value < 0.05 was considered statistically significant. However, given the exploratory nature of the study, findings should be interpreted cautiously and considered hypothesis-generating rather than confirmatory.

## Results

### Patient demographics and treatment characteristics

Forty-seven adult patients with bilateral vocal fold immobility were included in this retrospective cohort with a mean age of 43.3 ± 8.5 years, with a marked female predominance (93.6%). The most common aetiology was post-thyroidectomy injury (39 patients, 83%), followed by Hashimoto’s thyroiditis (8 patients, 17%). Most patients presented with bilateral vocal fold paralysis (87.2%), while 12.8% had bilateral paresis. All patients were non-tracheostomized. Dysphonia was reported in 38 patients (80.9%) prior to intervention. A staged botulinum toxin A (BTXA) injection protocol was used in all patients. The mean duration from symptom onset to BTXA injection was 3.07 ± 2.78 months. The onset of clinical effect was observed after a mean of 4.94 ± 0.70 days, with an average duration of effect of 3.74 ± 0.74 months. Patients received an average of 3.0 ± 0.96 injections during the study period. Demographic and clinical characteristics are summarized in Table 1.

**Table 1:** Patient demographics, clinical features, and treatment characteristics

Variable	Value
Age, mean (SD), y	43.3 ± 8.5
Sex	Male: 3 (6.4%) Female: 44 (93.6%)
Aetiology, No. (%)	Thyroidectomy: 39 (83%) Hashimoto’s: 8 (17%)
Diagnosis, No. (%)	Paralysis: 41 (87.2%) Paresis: 6 (12.8%)
Dysphonia before injection, No. (%)	38 (80.9%)
Duration since paralysis, mean (SD), mo.	3.07 ± 2.78
Onset of clinical effect, mean (SD), d.	4.94 ± 0.70
Duration of BTXA benefit, mean (SD), mo.	3.74 ± 0.74
Number of injections, mean (SD)	3.0 ± 0.96

Abbreviations; *VF*, vocal folds; *BTXA*, botulinum toxin type A.; *SD*, standard deviation. Values are presented as mean (SD) or number (%)

### Respiratory outcomes

**Fig. 3:**
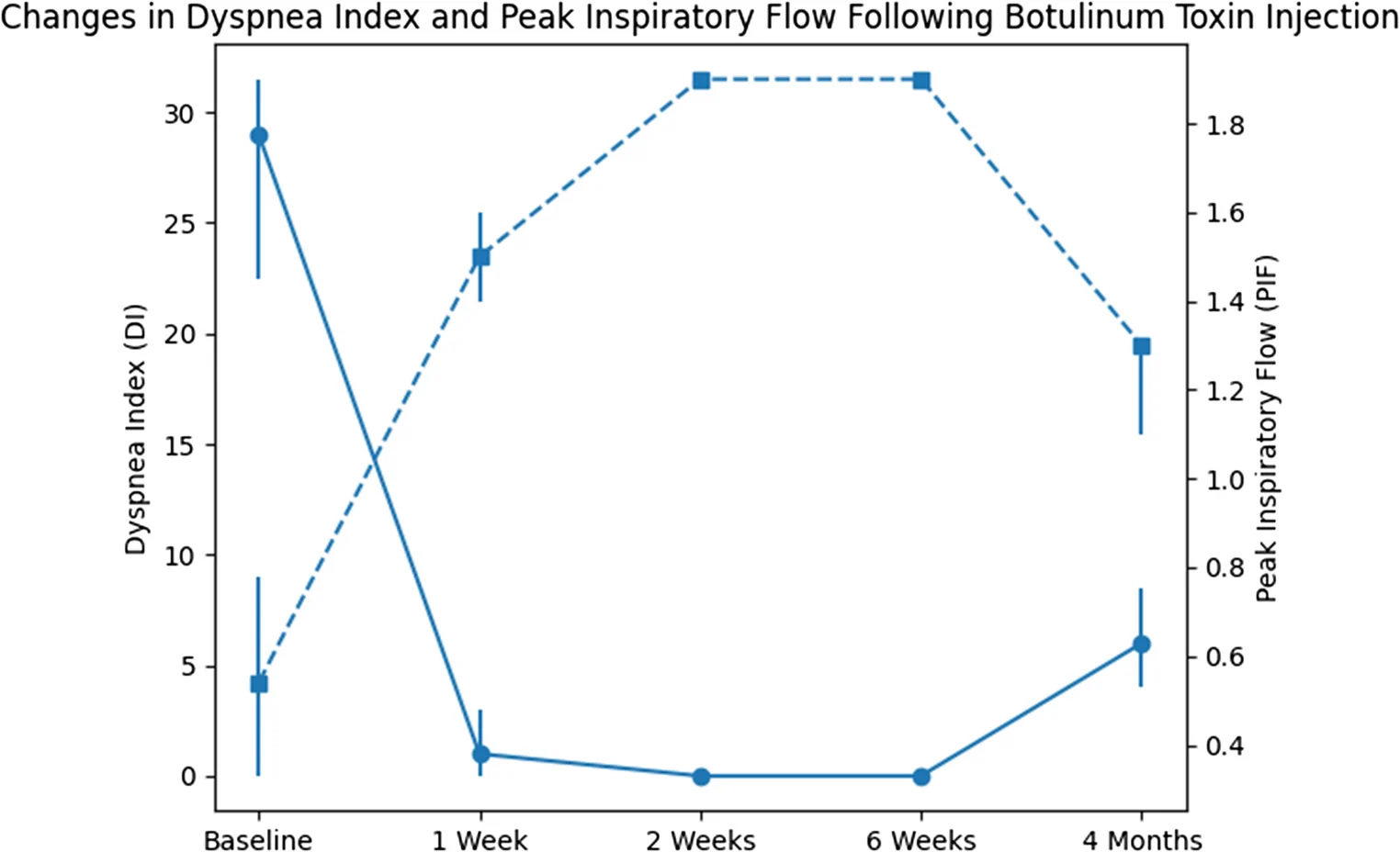
Respiratory outcomes before and after BTXA injection; DI decreased from a median of 29 pre-injection to 7 while, PIF increased from a baseline median of 0.54 to 1.3 at 4 months post injection, (*n* = 47). Error bars indicate mean ± SD

Dyspnea Index (DI) demonstrated a marked and statistically significant reduction at all post-injection time points compared with baseline (Wilcoxon signed-rank test, *p* < 0.05). Median DI decreased from 29 at baseline to 0 at both 2 and 6 weeks, indicating optimal clinical improvement, followed by a partial increase to 7 at 4 months consistent with the end of the pharmacological effect window. Peak inspiratory flow (PIF) showed a corresponding and significant increase, rising from a median of 0.54 at baseline to 1.9 at 2 and 6 weeks. Although a mild decline to 1.3 was observed at 4 months, PIF values remained higher than baseline levels (Fig. 3).

**Fig. 4:**
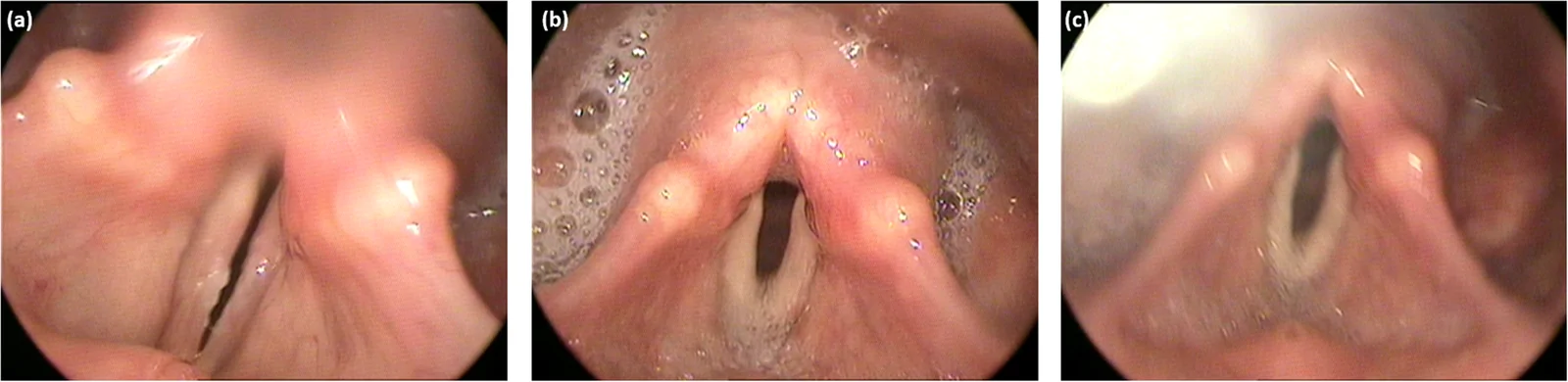
Laryngoscopic views of patient with bilateral vocal fold paralysis showing inspiratory chink changes pre- and post-BTXA injection: (**a**) pre-injection, (**b**) 1 week, (**c**) 2 weeks-post-injection, demonstrating airway widening. All images are anonymized and used with patient consent

Two patients developed respiratory distress shortly after the initial injection and subsequently underwent tracheostomy; both were decannulated two weeks after the second injection. Figure 4 shows glottal widening at 1 and 2 weeks post-injection. These observations are descriptive and occurred within the expected recovery window of early BVFI.

### Diagnosis and duration of benefit

There was no statistically significant difference in the duration of observed BTXA effect between patients with bilateral vocal fold paralysis and those with paresis (*p* = 0.138). Although the paresis group showed a slightly longer duration (4.17 ± 0.75 months) compared to the paralysis group (3.68 ± 0.72 months), the difference was not statistically significant. Given the small number of patients with paresis, this finding should be interpreted cautiously (Table 2).

**Table 2:** Association between diagnosis and duration (months) of BTXA benefit

Diagnosis	Number (%)	Mean ± SD	Median	Min-Max	*p*-value
Bilateral V.F Paralysis	41 (87.2%)	3.68 ± 0.72	4	3–5	0.138
Bilateral V.F paresis	6 (12.8%)	4.16 ± 0.75	4	3–5	

Values are presented as mean ± standard deviation. Mann-Whitney U test was used

### Temporal clinical outcomes following BTXA injection

Following BTXA injection, patients demonstrated a progressive improvement in dyspnea index (DI) scores over time, reflecting a reduction in respiratory symptoms. At 1 week post-injection, a noticeable improvement was observed, which became more pronounced at 2 weeks and reached its optimal level at 6 weeks, where DI scores were at their lowest. This pattern indicates a gradual and observed clinical benefit during the early post-treatment period. At 4 months, DI scores increased again, suggesting a partial decline in treatment efficacy over time, consistent with the known temporary effect of botulinum toxin. The peak clinical effect was observed at a mean of 10.31 ± 5.59 days, indicating a relatively rapid onset of action. Additionally, the duration of clinical benefit extended to a mean of 4.17 ± 0.20 months, demonstrating a sustained improvement in respiratory function for several months following injection. Overall, these findings highlight that BTXA injection provides time-dependent improvement in dyspnea, with a predictable clinical course characterized by early onset, peak effect within the first few weeks, and gradual waning over time (Table 3).

**Table 3:** Changes in Dyspnea Index and clinical outcomes following fractionated botulinum toxin injection

Treatment response	Mean (SD)
DI at 1 week	1.60 ± 1.62
DI at 2 weeks	0.26 ± 0.67
DI at 6 weeks	0.11 ± 0.31
DI at 4 months	9.98 ± 2.02
Peak clinical effect, d.	10.31 ± 5.59
Duration of benefit, mo.	4.17 ± 0.20

*SD*, standard deviation. Values are presented as mean (SD)

### Voice and swallowing outcomes

Voice and swallowing measures demonstrated transient early worsening followed by improvement during follow-ups. A significant deterioration at one week was observed across all parameters, particularly for dysphonia grade, VHI, and DHI, supported by moderate-to-large effect sizes, particularly for dysphonia grade (*r* = 0.62), VHI (*r* = 0.71), and DHI (*r* = 0.68), indicating a transient early post-injection effect. Subsequent assessments showed sustained improvement, with dysphonia grade and VHI decreasing below baseline levels. PAS scores showed a temporary increase (*r* = 0.59) at one week followed by non-significant differences at later assessments, suggesting no long-term impact on swallowing safety. DHI followed a similar trajectory, with early deterioration followed by significant improvement at four months (*r* = 0.63). Overall, these findings suggest a predictable short-term decline followed by sustained improvement, particularly in voice outcomes, without persistent swallowing impairment (Table 4).

**Table 4:** Voice and swallowing outcomes before and after BTXA injection (*n* = 47)

Measure	Pre-injection, (mean ± SD)	1 week	2 weeks	6 weeks	4 months	*p*-value*	Effect size (*r*)
Dysphonia grade	0.94 ± 0.63	2.43 ± 0.54	0.87 ± 0.58	0.51 ± 0.55	0.34 ± 0.48	< 0.001*, 0.412, < 0.001*, < 0.001*	0.62, 0.12, 0.55, 0.68
PAS score (FEES)	1.04 ± 0.20	2.11 ± 0.32	1.04 ± 0.20	1.06 ± 0.24	1.00 ± 0.00	< 0.001*, 1.000, 298, 168	0.59, 0.00, 0.10, 0.14
VHI score	7.57 ± 2.76	12.53 ± 3.63	7.66 ± 1.91	6.21 ± 1.36	4.94 ± 0.97	< 0.001*, < 0.001*, < 0.001*, < 0.001*	0.71, 0.52, 0.60, 0.75
DHI score	2.83 ± 1.72	7.34 ± 2.09	3.66 ± 1.94	2.77 ± 1.36	1.55 ± 1.29	< 0.001*, < 0.001*, 0.382, < 0.001*	0.68, 0.54, 0.11, 0.63

Abbreviations: *FEES*, Fiberoptic endoscopic evaluation of swallowing; *PAS*, Penetration–Aspiration Scale; *VHI*, Voice Handicap Index; *DHI*, Dysphagia Handicap Index. Values are presented as mean ± SD. P-values by Wilcoxon signed-rank test comparing each follow-up with baseline. Effect size (r) calculated as Z/√N. *p* < 0.05 considered statistically significant

### Correlation analysis

**Fig. 5:**
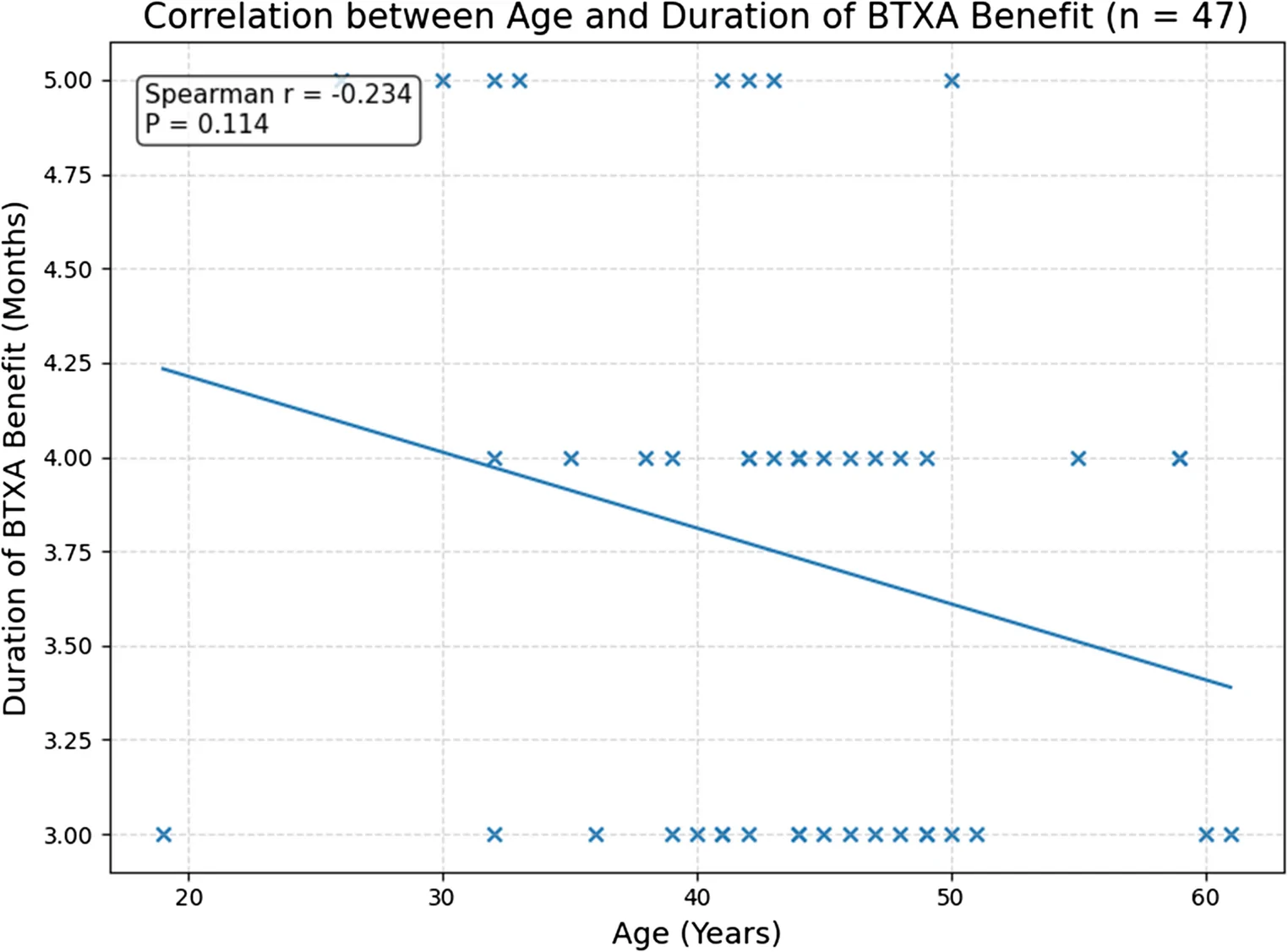
Scatter plot illustrating the relationship between patient age and duration of BTXA benefit (*n* = 47). Spearman correlation analysis demonstrated a weak negative, non-significant association between age and duration of therapeutic benefit (*r* = − 0.234, *P* = 0.114). The fitted line demonstrates the overall trend of the data

Patient age demonstrated a weak negative correlation with the duration of BTXA benefit; however, this association did not reach statistical significance (Spearman’s rank correlation, *r* = -0.234, *P* = 0.114), indicating no significant relationship between increasing age and duration of therapeutic effect in the analysed cohort (Fig. 5).

## Discussion

Airway compromise represents the principal clinical concern in early bilateral vocal fold immobility (BVFI). In the early phase of the disease, irreversible surgical interventions are often deferred due to the potential for spontaneous neurological recovery. Tracheostomy, while effective in securing the airway, is associated with significant morbidity and impact on quality of life [5]. Consequently, some patients opt to limit physical activity rather than undergo tracheostomy, which may further compromise functional status. Therefore, reversible and minimally invasive interventions that may temporize airway obstruction during the observation period remain of clinical interest.

Botulinum toxin type A (BTXA) reduces adductor muscle hyperactivity, potentially allowing reinnervated abductor fibres to act with less opposition and thereby increasing the inspiratory glottic aperture [6]. However, evidence supporting its use in BVFI remains limited to small observational series [9–11]. The present study describes a five-year single-centre clinical experience with awake, trans-oral intra-laryngeal BTXA injection in patients presenting with early-onset BVFI, focusing on feasibility, short-term airway changes, and peri-procedural outcomes within the expected pharmacological window of BTXA activity.

Patients in this cohort were treated during the early post-injury period, with a mean interval of approximately three months from symptom onset. Peripheral recurrent laryngeal nerve injuries in this context are typically neuropraxic rather than transectional, preserving axonal continuity and allowing for remyelination and functional recovery within the first four months after injury [4]. This timeframe corresponds to the observation window during which spontaneous improvement in vocal fold mobility may occur and provides a rationale for employing reversible, temporizing interventions rather than definitive surgical airway procedures.

Post-thyroidectomy injury accounted for the majority of cases in our series, reflecting local surgical practice patterns. In the absence of documented nerve transection or reanastomosis, the clinical presentation and postoperative course in our cohort are consistent with synkinetic reinnervation, often manifesting as paradoxical adductor activity during inspiration [22]. Although the paresis group showed a slightly longer duration of BTXA effect compared to the paralysis group consistently with previous series [23], there was no statistically significant difference. However, given the small size of the paresis group, this finding should be interpreted cautiously.

Intra-laryngeal BTXA injection was performed under endoscopic guidance rather than electromyography (EMG), reflecting resource availability. While EMG guidance is frequently used to confirm physiologic target activation, its contribution is primarily functional rather than anatomic [8–11]. Endoscopic visualization permits direct identification of laryngeal landmarks and inspiratory vocal fold movement, enabling practical and reproducible localization of the thyroarytenoid–lateral cricoarytenoid (TA–LCA) complex. Moreover, it reduces the risk of vocal oedema and ecchymosis sometimes observed with percutaneous EMG-guided injections [12].The injection site was selected based on anatomical validation by Alonso et al., who demonstrated diffusion of injected aniline dye from the TA into the adjacent LCA muscle [21]. Awake trans-oral injection under local anaesthesia was feasible in the outpatient setting and avoided the need for specialized equipment or general anaesthesia.

Improvements in Peak Inspiratory Flow (PIF) and Dyspnoea Index (DI) scores were observed shortly after injection and persisted throughout the follow-up period, with partial attenuation toward four months, consistent with the known pharmacological duration of BTXA [24]. However, given the absence of a control group and the known possibility of spontaneous recovery in early BVFI, these findings should be interpreted as temporal associations rather than evidence of treatment efficacy. Two patients experienced early postoperative respiratory distress requiring tracheostomy. Both subsequently improved clinically and were decannulated. These events are reported descriptively and should be interpreted cautiously in the context of early disease variability and recovery potential. Nevertheless, our observations are consistent with prior reports describing BTXA as a potential temporizing option for airway management in early-onset bilateral vocal fold immobility [10].

No standardized dose of BTXA has been established for bilateral vocal fold immobility (BVFI), and reported regimens vary widely [8–11]. A unilateral 10-U TA–LCA injection has shown favorable results in the study by Filho and Rosen [11]. However, because the TA and LCA muscles on both sides contribute to inspiratory glottic narrowing, bilateral chemodenervation is often required to achieve meaningful airway expansion. Accordingly, in this study we used a bilateral TA-LCA injection protocol to optimize airway patency based on prior clinical experience [19]. The TA adducts the membranous vocal fold, while the LCA adducts the arytenoid vocal process, and together they contribute to physiologic glottic closure [25].

The timing of clinical response and duration of observed effect were consistent with the known pharmacodynamic profile of botulinum toxin type A [26]. The occurrence of early post-injection airway events despite a 24-hour interval between injections suggests that such a short interval does not result in distinct temporal separation of the physiological effect between the two vocal folds. This observation supports prior pharmacological evidence regarding the onset and peak action of BTX-A rather than representing a distinct treatment effect, as airway-related improvements occurred within a timeframe similar to that reported for simultaneous bilateral injection [9, 10]. Rather, these findings suggest that bilateral BTXA injection in selected patients with BVFI is technically feasible and associated with short-term changes in airway-related measures. The two-session approach was introduced as a pragmatic procedural modification in patients with critically narrowed glottic apertures, based on concerns regarding transient airway compromise following bilateral injection [19]. The injection volume (0.2 mL saline aliquot) was typically resorbed within 24 h [27].

Dysphonia after bilateral vocal fold immobility, often presenting as a monotonous voice and short speech bursts due to compensatory stridor and disrupted vocal fold vibration [28]. In our study, voice and swallowing outcomes followed a predictable transient pattern, with early post-injection worsening followed by recovery and subsequent improvement. Temporary dysphonia and mild swallowing disturbances have been consistently reported after BTXA injection and were self-limited in this cohort [10, 29]. Importantly, no persistent swallowing impairment was detected on follow-up FEES, and patient-reported outcomes improved by four months. These findings support the tolerability of the procedure when patients are appropriately counselled regarding expected short-term effects.

Several limitations should be acknowledged. This was a retrospective, single-centre observational study with a relatively small sample size and clinical heterogeneity. The cohort included patients with varying degrees of vocal fold immobility and different aetiologies, which limits generalizability. The absence of a control group precludes any causal inference regarding treatment effect. Additionally, follow-up was limited to the expected duration of BTX-A action, and long-term outcomes beyond this period were not assessed. Therefore, the findings should be considered exploratory and hypothesis-generating. Despite these limitations, this series represents a relatively large single-centre experience describing the technical feasibility of intra-laryngeal BTX-A injection in early BVFI. The findings suggest that the procedure can be performed safely in an outpatient setting and may be associated with short-term changes in airway physiology during the observation period. However, further prospective controlled studies are required to define its role, optimal dosing strategy, and patient selection criteria.

## Conclusion

In this retrospective single-centre observational study, intra-laryngeal botulinum toxin type A injection was feasible in patients with early bilateral vocal fold immobility and was associated with short-term changes in airway-related physiological and symptom-based measures. Given the observational design and absence of a control group, these findings cannot be interpreted as evidence of treatment efficacy but rather as clinical and physiological observations within the early disease course, during a period in which spontaneous recovery may also occur. The study highlights the technical feasibility of awake trans-oral injection and provides real-world clinical experience in managing early BVFI in an outpatient setting. Further prospective controlled studies are required to define optimal dosing strategies, patient selection criteria, and the role of botulinum toxin within the overall management pathway of bilateral vocal fold immobility.

## Data Availability

The data supporting the findings of this study are included within the article. Additional data are available from the corresponding author upon reasonable request.
